# Immediate Implant-Based Breast Reconstruction Using TIGR® Matrix Surgical Mesh: Clinical Outcomes From Our First 100 Procedures

**DOI:** 10.7759/cureus.84745

**Published:** 2025-05-24

**Authors:** Giorgio Caddia, Mellie Heinemann, Marie Osdoit, Sylvie Chabaud, Léa Rossi, Christelle Faure, Sophie Klingler, Maria A Dammacco

**Affiliations:** 1 Plastic, Reconstructive, and Aesthetic Surgery Unit, Department of Human Pathology, Policlinico Universitario G. Martino, Messina, ITA; 2 Breast Unit, Department of Surgical Oncology, Centre Léon Bérard, Lyon, FRA; 3 Department of Clinical Research and Innovation, Centre Léon Bérard, Lyon, FRA

**Keywords:** breast cancer, implant-based breast reconstruction, mastectomy, synthetic mesh, tigr matrix

## Abstract

Background

Implant-based reconstruction has advanced with the introduction of acellular dermal matrices (ADMs). However, ADMs are associated with higher complication rates. Synthetic meshes have emerged as cost-effective alternatives. This retrospective, single-center study evaluates the clinical outcomes of immediate implant-based breast reconstruction with TIGR® Mesh (Novus Scientific, Uppsala, Sweden) on 76 patients (100 breasts) performed between February 2020 and June 2022.

Methods

Eligible patients were ≥18 years old with no major comorbidities and underwent direct-to-implant (DTI) mastectomy. Complications were assessed using the Clavien-Dindo classification (grade III or higher). Cosmetic outcomes were refined with lipofilling when required.

Results

The mean patient age was 45.5 years, with a mean follow-up of 17.3 months. Twelve mesh-related grade III complications were observed (10 infections and two seromas) over a total of 21 events. Implant loss occurred in 10 cases, which were later reconstructed in a subsequent procedure. No variable was identified as a significant predictor of complications. A significant inverse correlation (p=0.03) was found between mastectomy weight and complication rate, with lower weights associated with higher risk. Additionally, 57 reconstructions required at least one lipofilling session, with heavier mastectomy specimens needing fewer sessions.

Conclusions

TIGR® Mesh demonstrated an acceptable safety profile, with infection and seroma rates falling within the lower range reported for synthetic meshes and lower than most ADM series. Its cost-effectiveness and favorable outcomes support its use in selected patients. Further prospective studies are warranted to confirm these findings.

## Introduction

Breast reconstruction after mastectomy is a fundamental component of breast cancer treatment, contributing significantly to patients' psychological and physical well-being. The two main approaches are autologous tissue flaps and implant-based reconstructions. In the latter, the introduction of matrices, particularly acellular dermal matrices (ADMs), has revolutionized the field by improving implant support and aesthetic outcomes. ADMs are derived from either human (HADM) or animal (XADM) tissue.

While ADMs improve lower pole coverage and reduce capsular contracture, they are associated with high costs and complications such as seroma, infection, and implant loss, especially in the case of HADMs [1-3]. Synthetic meshes were developed to mitigate these issues, offering comparable structural support with potentially fewer complications and lower cost.

Among these, the TIGR® Matrix Surgical Mesh (Novus Scientific, Uppsala, Sweden) is a fully resorbable synthetic mesh composed of two distinct fiber types: a fast-resorbing copolymer of glycolide, lactide, and trimethylene carbonate, which integrates into the tissue within four months, and a slow-resorbing copolymer of lactide and trimethylene carbonate, which is absorbed over three years. These properties enable TIGR® Mesh to offer temporary mechanical support while progressively integrating into the tissue over a three-year period [4,5].

This study aims to report the clinical outcomes of immediate implant-based breast reconstruction using TIGR® Mesh, based on our early experience.

## Materials and methods

We designed a retrospective, single-center, multi-surgeon study on our cohort of implant-based immediate breast reconstruction utilizing the TIGR® Mesh.

The inclusion criteria for the use of TIGR® Mesh were as follows: age over 18 years and patients undergoing monolateral or bilateral direct-to-implant (DTI) mastectomy for oncological or prophylactic reasons.

Exclusion criteria included the inability to provide informed consent, severe obesity (BMI>40), and diabetes mellitus. Additionally, patients undergoing two-stage breast reconstruction were excluded and removed from the study database.

As a result, our database included 76 patients who underwent surgery between February 2020 and June 2022, accounting for a total of 100 breast reconstructions. All patients were assessed by a multidisciplinary team prior to surgery.

Surgical complications were assessed using the Clavien-Dindo classification system, focusing on those classified as grade III or higher [6].

Surgical technique

All patients were operated on by senior surgeons of a large team. The same surgeon performed both the mastectomy and reconstruction.

Prophylactic antibiotics were administered at the induction of anesthesia. Following asepsis with a betadine solution and surgical draping, the subcutaneous infiltration of saline with adrenaline (1 mg/500 mL) was systematically performed. Mastectomy was carried out using scissors, gliding along the ridges of Duret.

**Figure 1:**
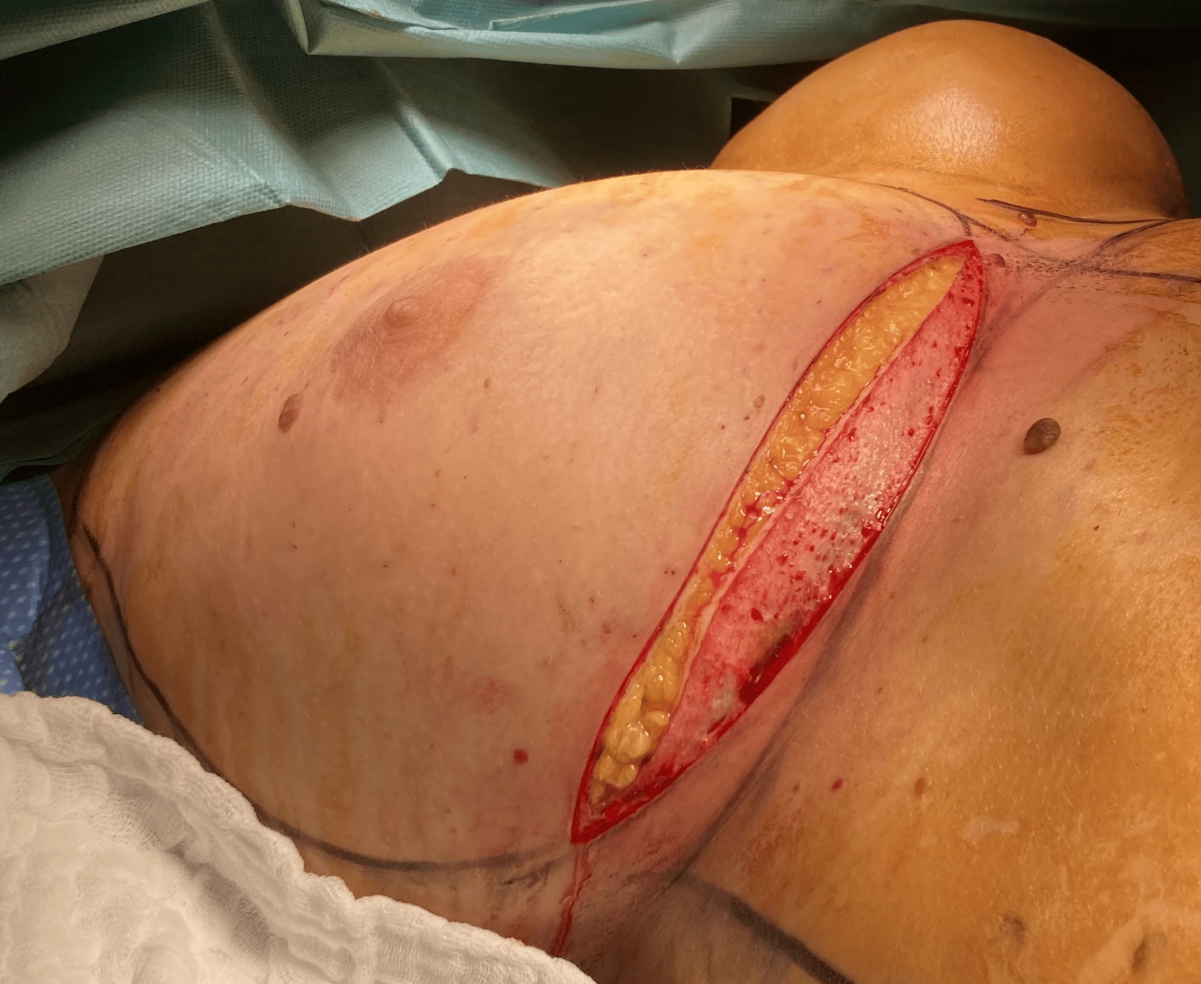
An intraoperative picture of an IMF access with the elliptical de-epidermized area used to preserve the lower pole of the reconstruction IMF: inframammary fold

In cases involving an inframammary fold (IMF) access, the de-epidermization of an elliptical area was performed to preserve the lower pole of the reconstruction (Figure 1).

**Figure 2:**
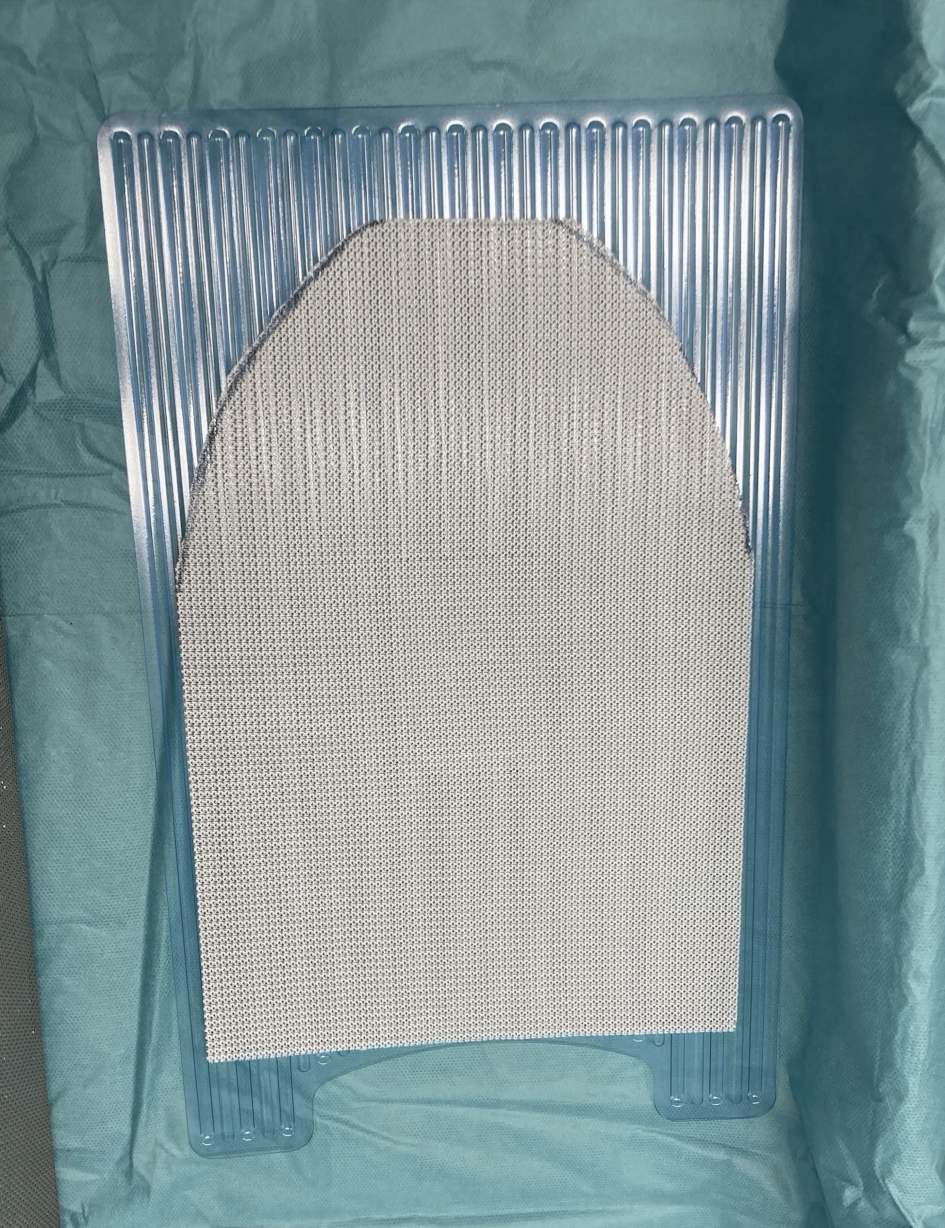
The TIGR® Mesh shaped into an ogive at its superior edge

**Figure 3:**
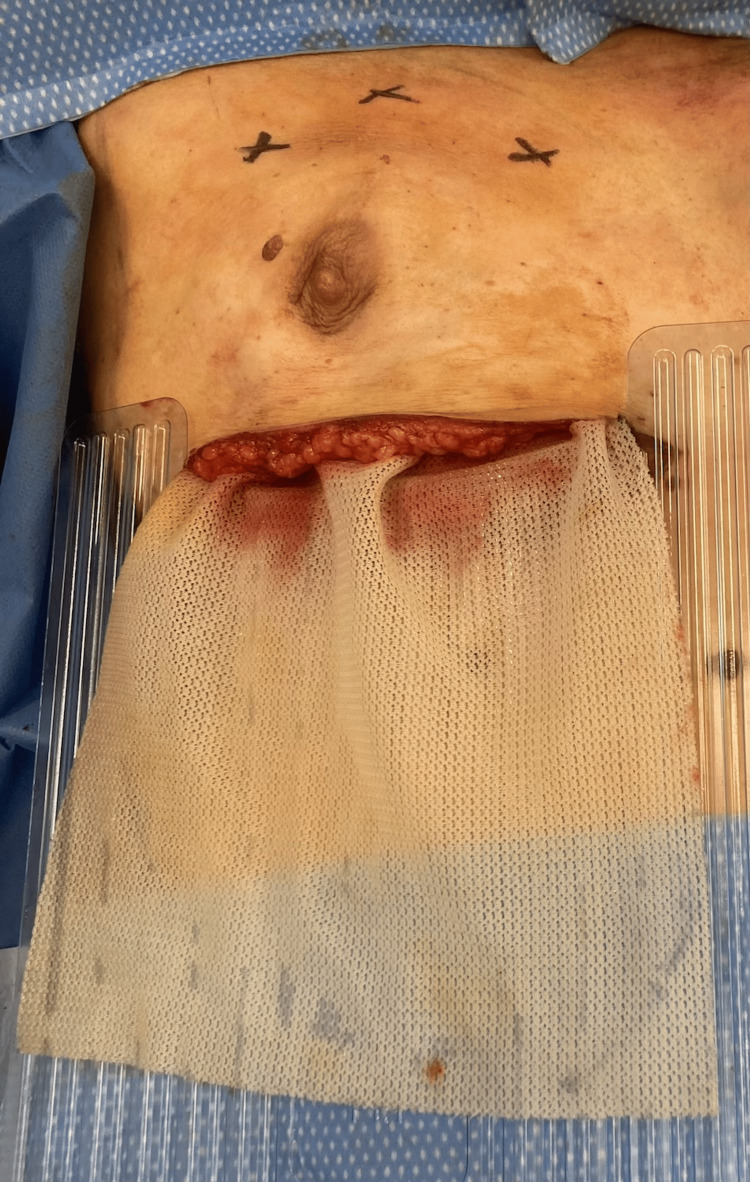
The TIGR® Mesh superior edge insetting Note the cutaneous projection of the muscular anchoring sutures marked as an "x" on the skin of the upper pole

**Figure 4:**
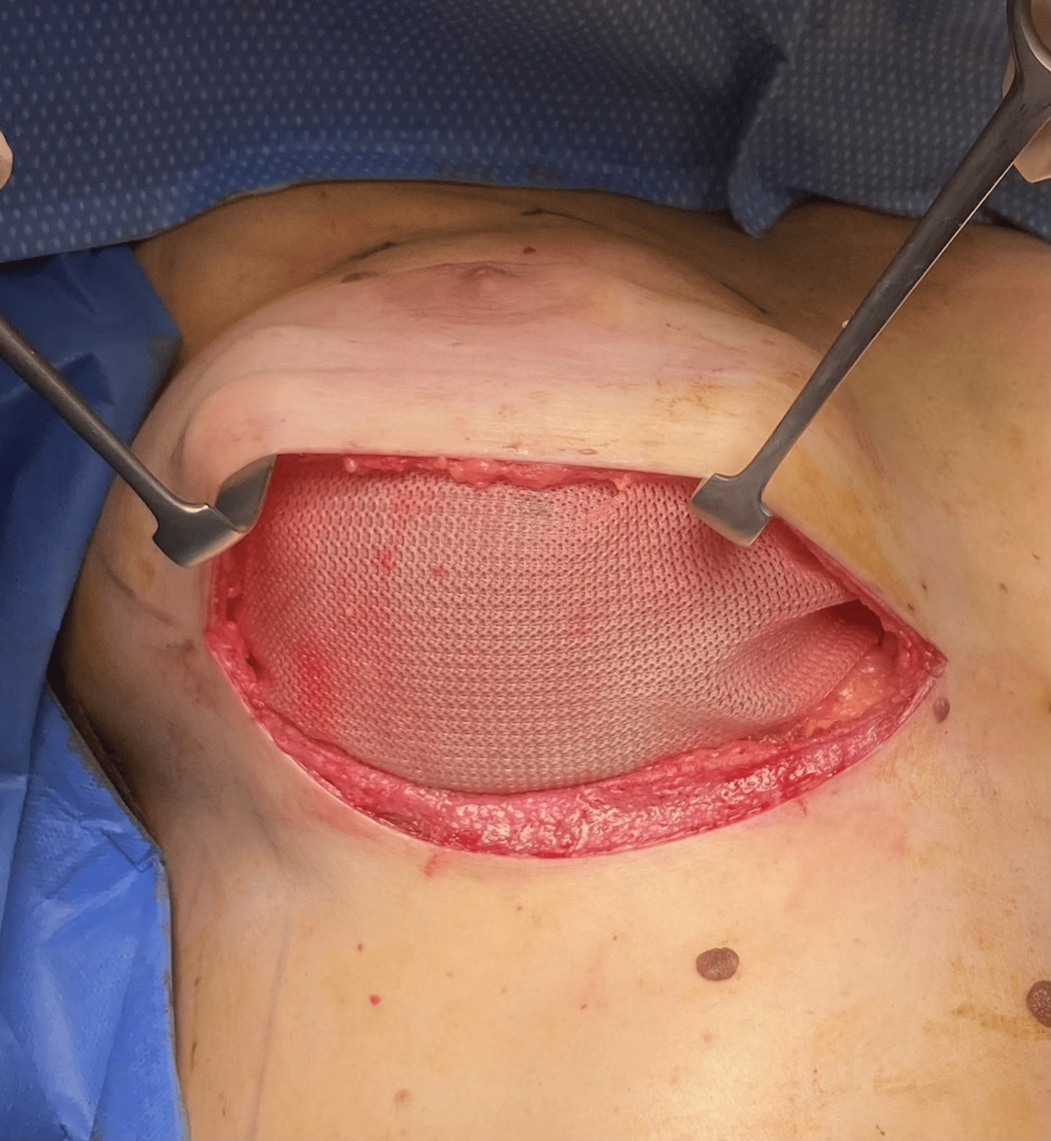
The final insetting of the implant and hammock-shaped TIGR® Mesh in prepectoral position

For the prepectoral approach, the TIGR® Mesh was shaped into an ogive at its superior edge (Figure 2) and secured to the pectoralis muscle at the upper pole of the pocket using medial, meridian, and lateral stitches (Vicryl® 2/0, Ethicon, Inc., Raritan, NJ) (Figure 3). After implant placement, the lower portion of the mesh supported the implant within a hammock-shaped pocket, ensuring the mesh was evenly distributed to prevent excess folds (Figure 4).

**Figure 5:**
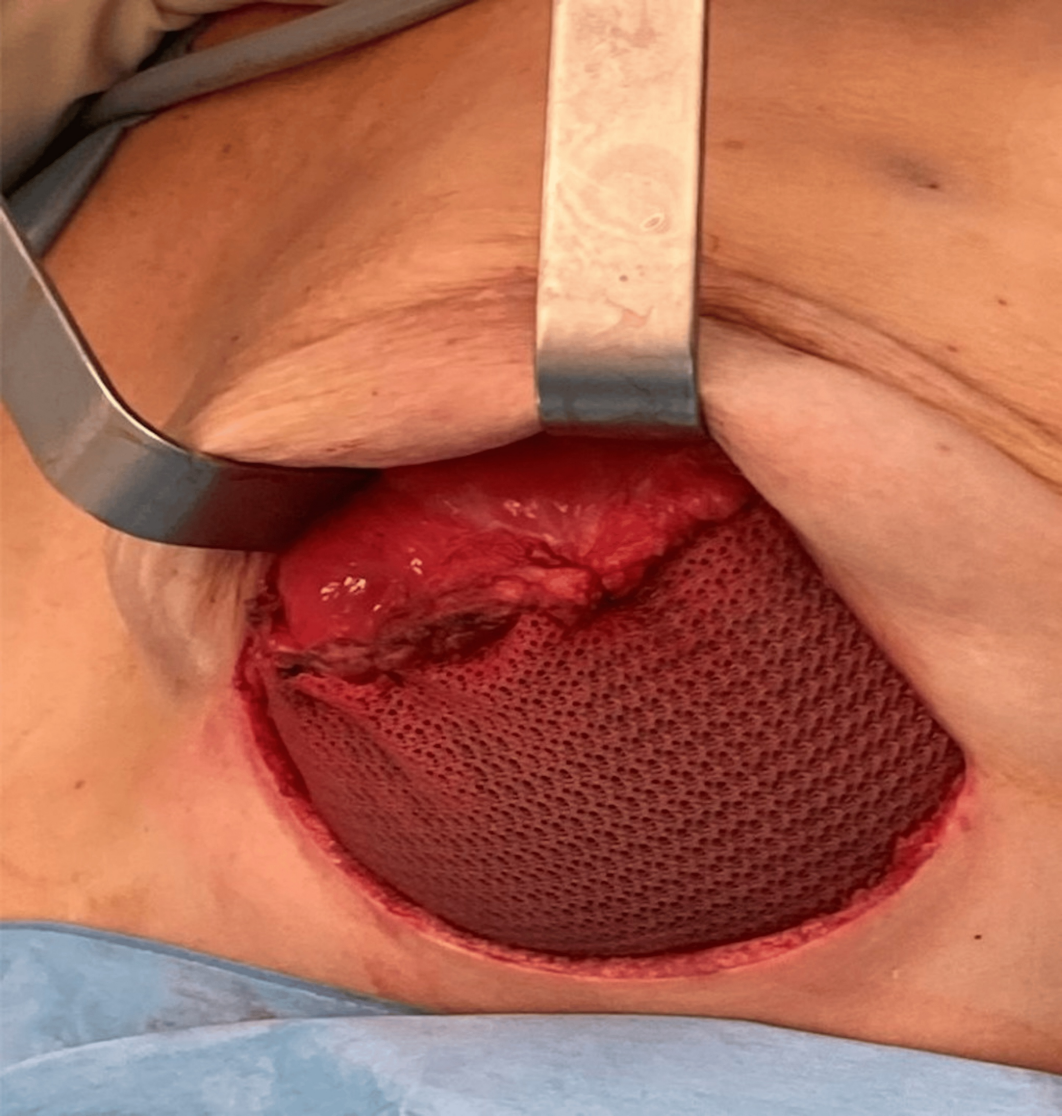
The final insetting of the implant and TIGR® Mesh in retropectoral position

For the retromuscular approach, a subpectoral pocket was created by suturing the mesh to the inferiorly detached pectoralis muscle (Figure 5).

**Figure 6:**
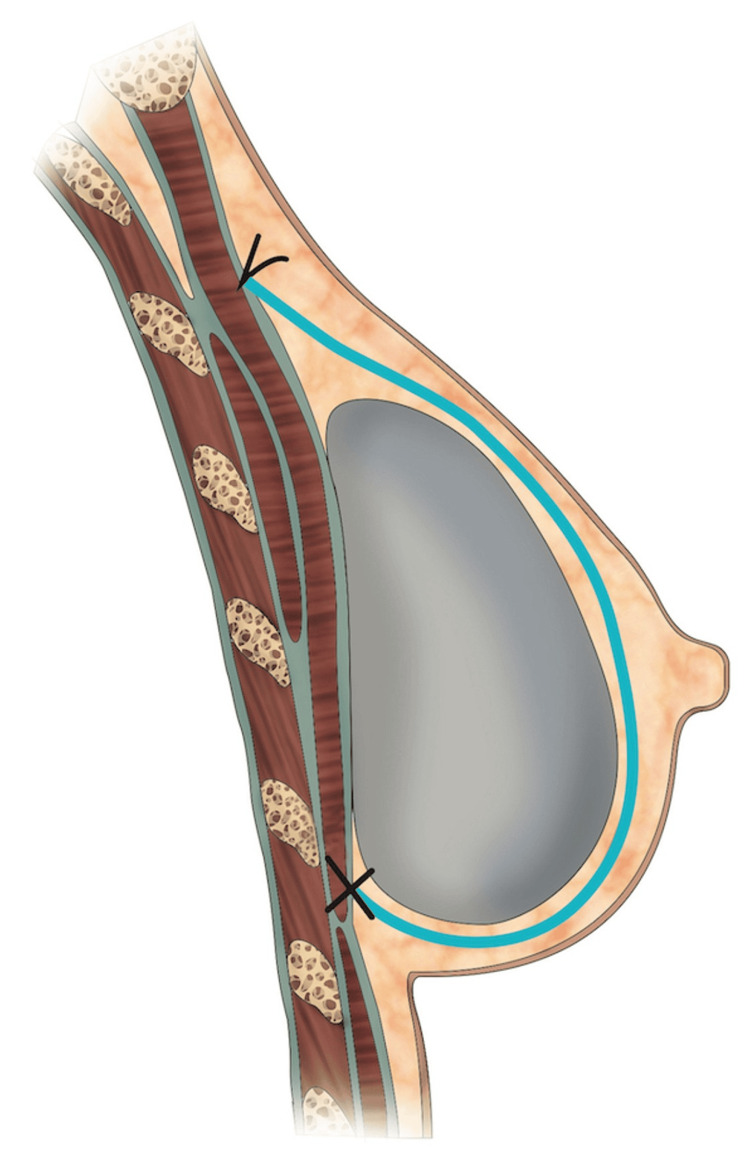
A visual representation of the sagittal view of implant-based breast reconstruction with TIGR® Mesh (blue line) in the prepectoral position Original illustration created by Marta Arca expressly for inclusion in this manuscript. Used with permission

**Figure 7:**
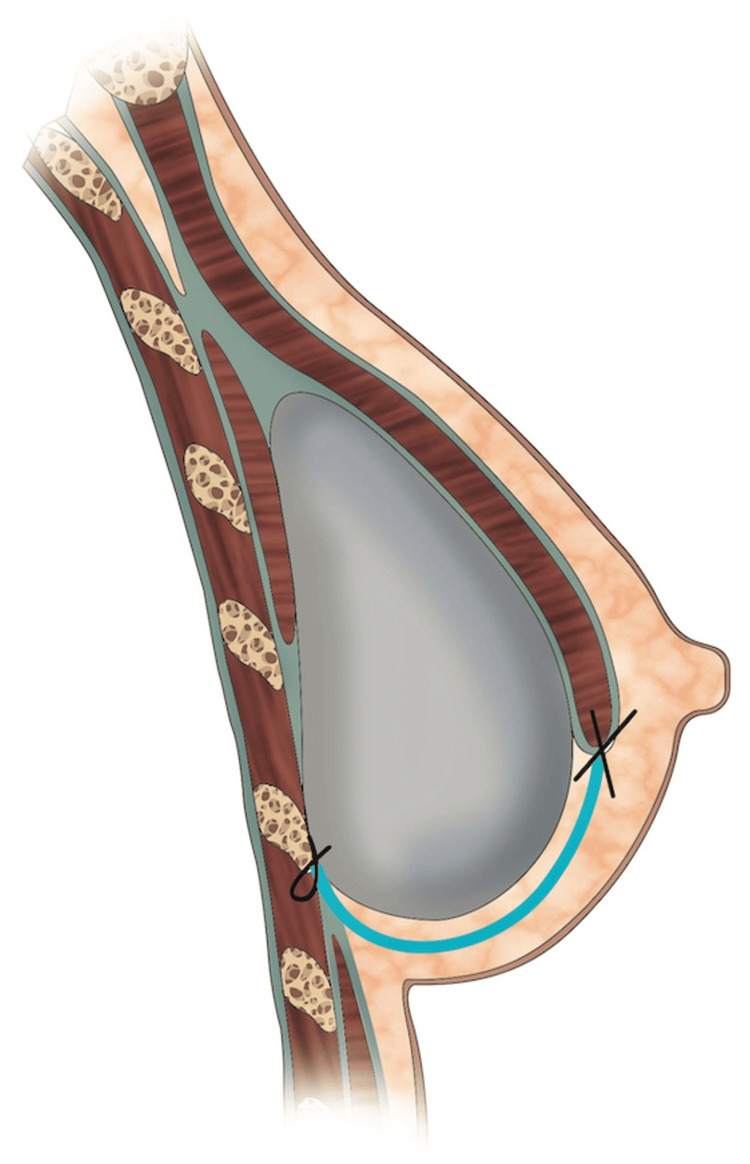
A visual representation of the sagittal view of implant-based breast reconstruction with TIGR® Mesh (blue line) in the retropectoral position Original illustration created by Marta Arca expressly for inclusion in this manuscript. Used with permission

Figure 6 and Figure 7 provide a visual representation of the differences in the insetting of the TIGR® Mesh between the two reconstruction planes.

The implants used in all cases were round, smooth implants (Motiva®, Establishment Labs, Alajuela, Costa Rica). Finally, drains were placed in the pocket, and surgical closure was achieved in two layers using Monocryl® sutures (Ethicon, Inc., Raritan, NJ). The mean duration of surgery was 134.8 minutes (range: 55-330 minutes).

**Figure 8:**
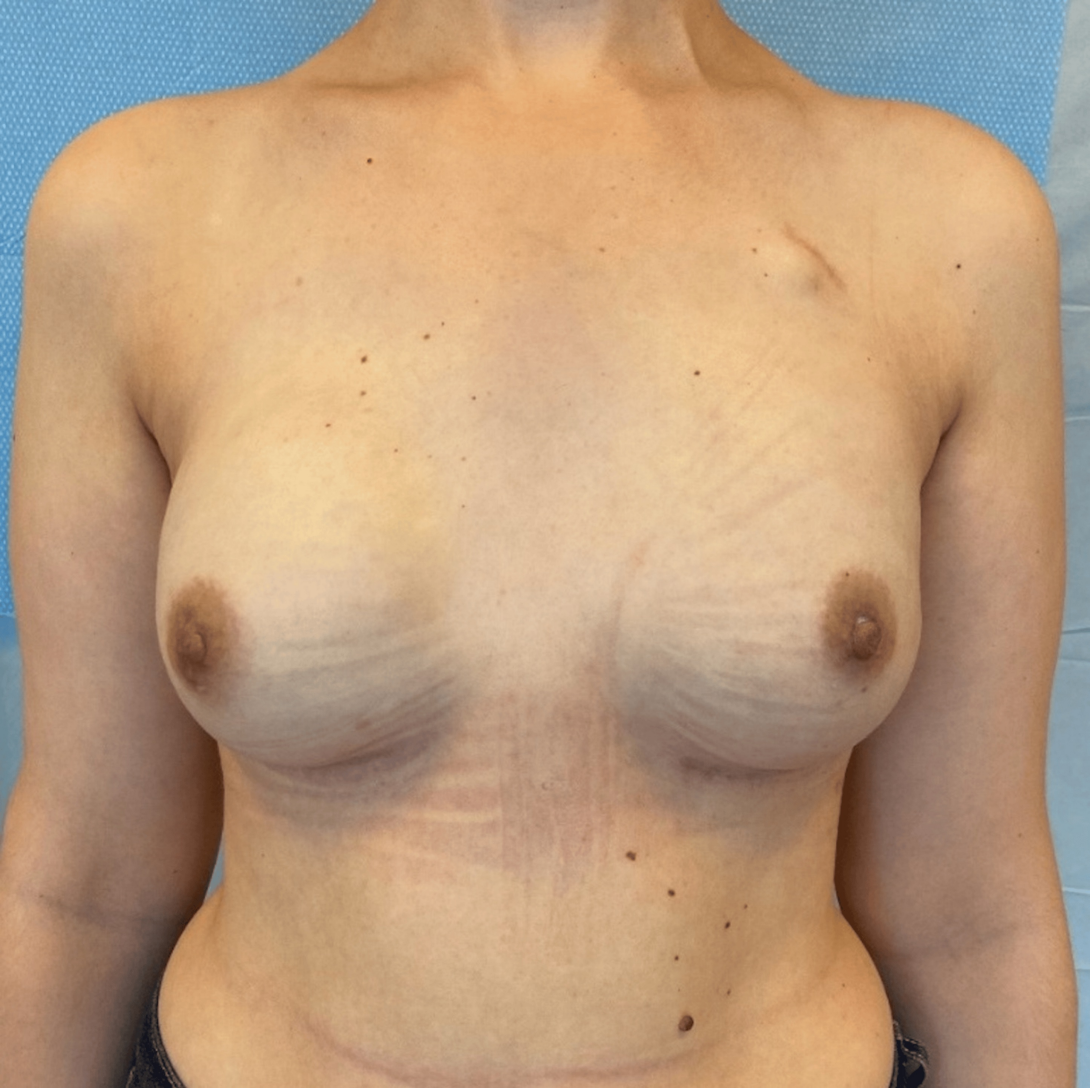
A photograph of a patient one month after undergoing bilateral mastectomy and reconstruction with implant and TIGR® Mesh

The initial follow-up clinical evaluation typically occurred between the second and third postoperative week, followed by a second evaluation at one month (Figure 8). Subsequent visits were scheduled individually, tailored to each patient's specific needs. The mean follow-up period was 17.7 months (range: 2-41 months).

Statistics

Each procedure was considered as the statistical unit, which means that if several procedures were performed on the same patient, they would be considered independent in terms of patient, surgical, and complication characteristics.

The statistical evaluations have been performed by using the software package Statistical Analysis System (SAS) version 9.4 (SAS Institute Inc., Cary, NC).

The descriptive analysis of the data has been performed using summary statistics for categorical and quantitative (continuous) data. Continuous data have been described by the number of non-missing values, median, mean, standard deviation, minimum, and maximum. Frequency tables have been generated for categorical data. Comparison between groups was done using the Wilcoxon nonparametric test for continuous data and Fisher's exact test for categorical data. A nominal p value of 0.05 was considered significant.

## Results

During the study period, a total of 100 breast reconstructions utilizing TIGR® Mesh were performed on 76 patients, with a mean age of 46.1 years (range: 24-74 years). The characteristics of the patients are summarized in Table 1. Twenty-four patients had a bilateral procedure.

**Table 1:** Cohort characteristics

Variable	Value
Number of patients	76
Mean age, years (range)	45.5 (24-74)
BMI (range)	23.2 (15-37)
Smokers	16
Bilateral	44
Previous breast surgery	35
Previous radiotherapy	12
Neoadjuvant chemotherapy	14
Mean mastectomy weight, g (range)	361.8 (40-770)
Radiotherapy	16
Adjuvant chemotherapy	18
Hormonotherapy	38
Follow-up, m (range)	17.3 (2-41)

The surgical specifics are summarized in Table 2. The reason for the mastectomy was a primitive cancer in 47 cases, local recurrence in seven cases, and *BRCA* mutation (prophylactic mastectomy) in 46 cases. Of all mastectomies, 81 were nipple-sparing (NSM) and 19 were skin-sparing (SSM). The preferential surgical approach was through the inframammary fold (IMF, 76 cases).

**Table 2:** Surgical specifics NSM, nipple-sparing mastectomy; SSM, skin-sparing mastectomy; IMF, inframammary fold; SLN, sentinel lymph node; LND, lymph node dissection

Variable	Category	n
Indication	Primitive cancer	47
	Local recurrence	7
	*BRCA* mutation	46
Type of mastectomy	NSM	81
	SSM	19
Incision	IMF	76
	Emi-periareolar	2
	Radial	2
	Periareolar involving nipple removal	20
Position of the implant	Prepectoral	76
	Retropectoral	24
Concomitant axillary surgery	No	50
	SLN	39
	LND	11

Within the first 90 postoperative days, 21 grade III complications were observed in 18 patients. No grade IV or V complications were reported. The results are summarized in Table 3. Twelve of these mastectomies were done for prophylactic reasons, eight for primitive cancer, and one for local recurrence. The complications included hematomas (n=7), infections (n=10), wound dehiscence (n=2), and seromas (n=2). In addition, we report three cases of red breast syndrome (RBS). Notably, no significant mastectomy flap necrosis or nipple-areola complex (NAC) necrosis was observed. Of these complications, 16 occurred in NSM cases and five in SSM cases, with 18 complications observed in the prepectoral group and three in the retropectoral group. Notably, these complications were distributed as follows: 14 NSM, prepectoral; two NSM, retropectoral; four SSM, prepectoral; and one SSM, retropectoral. Of the total cases, 10 resulted in implant loss; these patients were initially left without reconstruction and later underwent secondary reconstruction in a subsequent surgical procedure. The mean weight of mastectomy in cases that had a complication was 297.1 g, with lower values observed in cases involving hematomas and seromas. None of these patients received postoperative chemotherapy. Three patients underwent postoperative radiotherapy, with one experiencing a significantly delayed initiation of radiotherapy (23 weeks).

**Table 3:** Complications NSM, nipple-sparing mastectomy; SSM, skin-sparing mastectomy

Variable	Hematoma	Infection	Seroma	Wound dehiscence	Total
NSM	5	8	1	2	16
SSM	2	2	1	0	5
Prepectoral	5	9	2	2	18
Retropectoral	2	1	0	0	3
Mean mastectomy weight (g)	237.14	341.3	292	384	297.1

**Figure 9:**
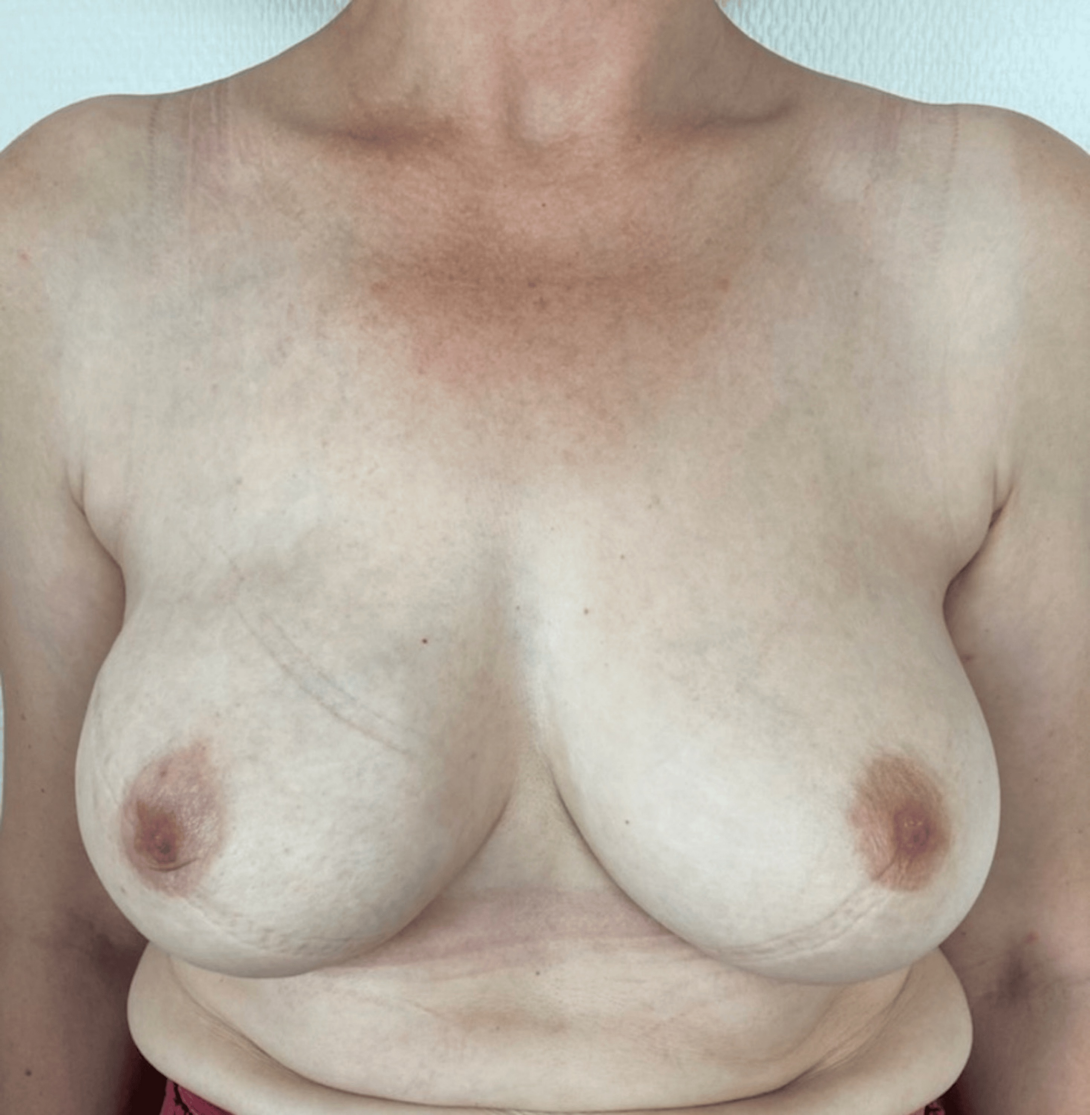
A photograph of a patient one month after undergoing right breast reconstruction with implant and TIGR® Mesh in prepectoral position

**Figure 10:**
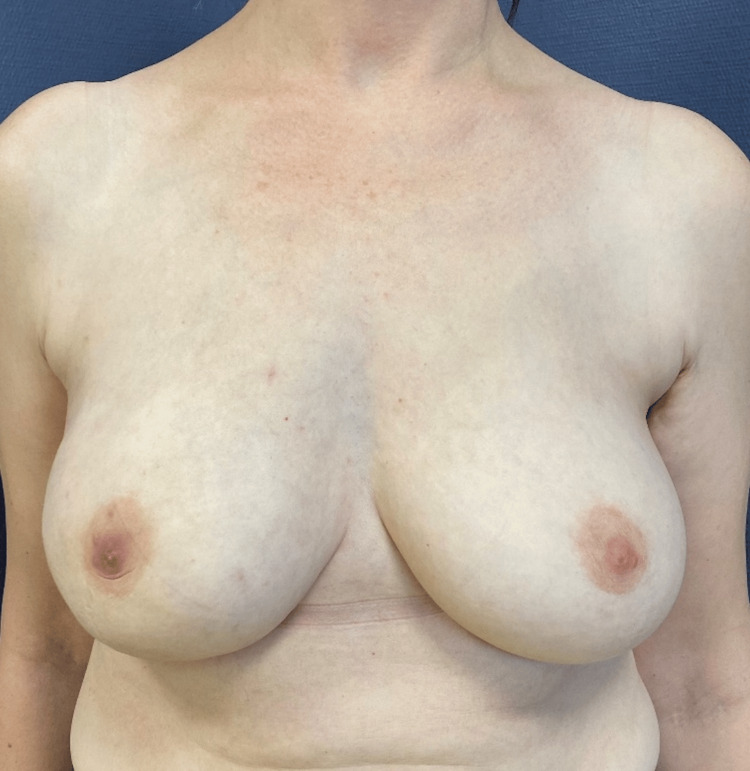
The same patient of Figure 9 after undergoing one session of lipofilling

Regarding refinements with lipofilling, 57 of our 100 reconstructed breasts required at least one session of lipofilling to enhance the cosmetic outcome of the reconstruction (Figure 9 and Figure 10). Among these, 45 reconstructions required only one session, while 12 required two or more sessions. The majority of these cases (42 out of 57) involved prepectoral reconstructions.

## Discussion

The overall complication rate in our cohort was 21%, with 10 implant losses. Although this rate is slightly higher than those reported in previous studies using synthetic meshes, it remains within an acceptable clinical range and reflects real-life practice, including prophylactic and oncological cases without strict exclusion criteria [7-11].

We observed no significant correlation between complications and age (p=0.559), BMI (p=0.545), smoking habit (p=0.32), history of homolateral breast surgery (p=0.81), and duration of surgery (p=0.509).

Among subgroups, the NSM and prepectoral approaches showed slightly higher complication rates, but these differences were not statistically significant.

Interestingly, complications were more frequent in patients with lower mastectomy weights (297.1 g versus 379.0 g; p=0.03), particularly for hematoma and seroma. These findings are inconsistent with the existing literature, as some studies support our observations [12], while others associate higher mastectomy weights with increased complication rates [13].

To simplify the presentation of our results regarding complications, we followed the approach of Pompei et al., who categorized complications into "surgery-related" (hematoma and wound dehiscence) and "mesh-related" (seroma and infection) [7]. Based on this classification, we observed nine surgery-related complications and 12 mesh-related complications.

When comparing ADMs and synthetic meshes, it is essential to consider clinical outcomes, cost-effectiveness, and patient satisfaction. Given the lack of randomized data directly comparing XADM to synthetic meshes and considering their comparable complication rates, our discussion will focus on HADMs, referred to here simply as ADMs [3].

Studies indicate that ADMs in breast reconstruction are associated with a higher rate of infection (between 0.2% and 35.8%) compared to synthetic meshes (between 1.3% and 6.1%) [3,10,11]. Our infection rate of 10% slightly exceeds typical values for synthetic meshes, potentially due to our protocol excluding postoperative antibiotics.

The higher rates of infection with ADMs may be due to biofilm-producing *Staphylococcus aureus*, which shows a predilection for biological materials [14] and the increased incidence of seroma: reported rates for synthetic meshes typically range from 0% to 5.7%, while seroma rates for ADMs are between 1.5% and 24.3% [9,10].

In our study, the seroma rate was 2%, falling within the lower range reported for synthetic meshes. The literature does not report significant differences in complication rates when comparing prepectoral and retropectoral approaches [15].

The higher seroma rate in ADMs may be related to their smooth surface, which promotes tissue movement, while the rougher texture of synthetic meshes allows for faster integration and reduced seroma formation [9,16].

Reported implant loss rates range from 0% to 8.7% for both ADMs and synthetic meshes [9,10,17]. Our implant loss rate was 10%, slightly above this. However, this result should be interpreted within the context of a heterogeneous, nearly unselected population, including both complex oncological and prophylactic cases, thus reflecting real-world clinical practice.

Capsular contracture was observed in 3% of cases at six months, consistent with literature data (0.4%-8.6%) [9,10]. Regarding patient satisfaction, studies show no significant difference between ADMs and synthetic meshes. However, complications and the impact of radiotherapy remain major concerns for patients [9,18].

Lipofilling is essential to achieve optimal cosmetic outcomes [19]. In our series, 57% of reconstructions required at least one session. A significant inverse correlation (p=0.005) was observed between mastectomy weight and the number of lipofilling sessions needed.

The main strength of TIGR® Mesh lies in its progressive resorption and biocompatibility, which provide adequate mechanical support during healing while avoiding long-term foreign body presence. Additionally, it is significantly more affordable than ADMs ($3,000 for a 15 × 20 cm piece of TIGR® versus $9,000 for a comparable piece of AlloDerm). In resource-constrained environments, this cost-effectiveness can expand access to immediate implant-based reconstruction without compromising outcomes [1,3,10,11].

The main limitations of this study are its retrospective nature and the absence of a direct internal control group. Comparative analysis was therefore based on literature data, which may be affected by heterogeneity in surgical techniques, team expertise, and institutional settings.

## Conclusions

Our experience with TIGR® Matrix Surgical Mesh in immediate implant-based breast reconstruction suggests that it is a safe and cost-effective alternative to traditional ADMs. Despite a slightly higher implant loss rate (10%), the overall complication rates, including infection and seroma, remained within acceptable limits and consistent with data from previous studies on synthetic meshes. We observed a significant inverse correlation between mastectomy weight and both complication rates and the number of lipofilling sessions required, although the biological plausibility of these findings remains to be confirmed.

The main advantages of TIGR® Mesh lie in its progressive resorption, biocompatibility, and lower cost, which may broaden access to immediate reconstruction in resource-constrained settings and selected patient populations. However, the retrospective design, absence of a control group, and presence of multiple uncontrolled variables limit the generalizability of our conclusions. Future prospective, randomized studies with larger cohorts are necessary to validate these findings and better define the role of synthetic resorbable meshes in breast reconstruction.
